# Three New Structures of Left-Handed RadA Helical Filaments: Structural Flexibility of N-Terminal Domain Is Critical for Recombinase Activity

**DOI:** 10.1371/journal.pone.0004890

**Published:** 2009-03-19

**Authors:** Yu-Wei Chang, Tzu-Ping Ko, Chien-Der Lee, Yuan-Chih Chang, Kuei-Ann Lin, Chia-Seng Chang, Andrew H.-J. Wang, Ting-Fang Wang

**Affiliations:** 1 Institute of Biochemical Science, National Taiwan University, Taipei, Taiwan; 2 Institute of Biological Chemistry, Academia Sinica, Taipei, Taiwan; 3 Institute of Physics, Academia Sinica, Taipei, Taiwan; 4 Institute of Molecular Biology, Academia Sinica, Taipei, Taiwan; Tel Aviv University, Israel

## Abstract

RecA family proteins, including bacterial RecA, archaeal RadA, and eukaryotic Dmc1 and Rad51, mediate homologous recombination, a reaction essential for maintaining genome integrity. In the presence of ATP, these proteins bind a single-strand DNA to form a right-handed nucleoprotein filament, which catalyzes pairing and strand exchange with a homologous double-stranded DNA (dsDNA), by as-yet unknown mechanisms. We recently reported a structure of RadA left-handed helical filament, and here present three new structures of RadA left-handed helical filaments. Comparative structural analysis between different RadA/Rad51 helical filaments reveals that the N-terminal domain (NTD) of RadA/Rad51, implicated in dsDNA binding, is highly flexible. We identify a hinge region between NTD and polymerization motif as responsible for rigid body movement of NTD. Mutant analysis further confirms that structural flexibility of NTD is essential for RadA's recombinase activity. These results support our previous hypothesis that ATP-dependent axial rotation of RadA nucleoprotein helical filament promotes homologous recombination.

## Introduction

Homologous recombination is a ubiquitous mechanism for maintaining genome integrity and also for generating genetic diversity in sexual reproductive organisms. This reaction is catalyzed by RecA family proteins, including bacterial RecA, archaeal RadA, and eukaryal Rad51 and Dmc1. The current model holds that, in the presence of ATP, the recombinases coat a primary single-stranded DNA (ssDNA) to form a nucleoprotein right-handed helical filament, and initiate a search for a secondary homologous stretches of double-stranded DNA (dsNDA). The ssDNA then invades and displaces the homologous strand in the donor dsDNA, resulting in a new heteroduplex (or D-loop). Eventually, the homologous ssDNA will be expelled from the nucleoprotein filament [1], [2], [3].

*Escherichia coli* RecA (*Ec*RecA) is the founding member of the RecA protein family. It contains three major structural domains: a small N-terminal domain (NTD), a catalytic domain (CAD) and a large C-terminal domain (CTD). The CAD, often referred to as the RecA fold [4], is structurally similar to the ATPase domains of DNA/RNA helicases, F1 ATPases, chaperone-like ATPases, and membrane transporters [5]. The CAD contains two disordered loops (the L1 and L2 motifs) that bind to ssDNA and are responsible for the ssDNA-stimulated ATPase activity [6]. Two positively-charged CAD residues, Arg243 and Lys245, are responsible for binding to donor dsDNA [7], [8], [9], [10]. The CTD may also have a similar function in the RecA-ssDNA nucleoprotein filament of capturing donor dsDNA [7], [8]. RecA polymerization is mediated by the polymerization motif (PM) that is located between the NTD and the CAD. PM contains a hydrophobic residue (i.e., Ile26) that docks within the hydrophobic pocket of the neighboring CAD. Nikola Pavletich and colleagues recently reported the crystal structures of *Ec*RecA-ssDNA and *Ec*RecA-dsDNA nucleoprotein complexes with Mg^2+^, ADP and AlF_4_^−^ [11]. These right-handed filament structures have provided unprecedented new insights into the mechanisms and energetic of *Ec*RecA. [11], [12]. Here, ADP-AlF_4_^−^ was used to mimic the ADP-Pi, because AlF_4_^−^ is able to substitute for inorganic phosphate (Pi) after the hydrolysis of ATP. The *Ec*RecA-ssDNA-Mg^2+^-ADP-AlF_4_^−^ nucleoprotein filament represents the structural intermediate responsible for homology pairing to a donor dsDNA. By contrast, the RecA-dsDNA-ADP-AlF_4_^−^-Mg^2+^ crystal structure was postulated to be an end product after strand exchange reaction between RecA-ssDNA nucleoprotein filament and a homologous dsDNA target, implying that RecA protein filaments may complete all functions (including ssDNA binding, donor dsDNA capturing and strand exchange) within the axes of right-handed filaments. Here we consider an alternative possibility that the RecA-dsDNA crystal structures might simply represent annealing products of the ssDNA in RecA-ssDNA nucleoprotein filament and a complementary ssDNA. Firstly, in the RecA-ssDNA filament structure, the purine and pyrimidine bases of bound ssDNA are outwardly exposed. Secondly, the complementary ssDNA in the RecA-dsDNA structure makes very few physical contacts with RecA protein filament, indicating that the annealing of these two ssDNAs has very little impact on the protein structures. The overall protein structures of RecA-dsNDA filaments are highly similar to those of RecA-ssDNA-ADP-AlF_4_^−^-Mg^2+^ structures [11]. Because the molecular mechanism of homology pairing and strand exchange reaction is still not understood, it is important to further examine these two different possibilities.

*Ec*RecA is structurally and functionally different from eukaryotic Rad51 and Dmc1 proteins. By contrast, archaeal RadA is a better model for eukaryotic recombinases. First, the amino acid sequences of RadA proteins are highly conserved with those of Rad51 and Dmc1. The NTDs (>60 amino acid residues) of archaeal and eukaryotic proteins are similar in size, while the corresponding region of *Ec*RecA contains only 24 amino acid residues. Second, the NTDs of the human Rad51 and the arcaheal RadA protein have both been implicated as the dsDNA binding domain [9], [22] while RecA, in contrast, has an extra CTD for dsDNA binding. Finally, eukaryotic Rad51 and Dmc1 are known to interact with several mediator proteins, including Rad52, Rad54, Rad55/57, Brca2, Hop2-Mnd1, *etc.* [3]. An archaeal paralog of Rad55 has recently been isolated and characterized [13].

As part of the on-going investigation of the structure-function relationships of archaeal and eukaryotic proteins, we and other investigators had reported several crystal structures for RadA/Rad51/Dmc1 polymers, including protein rings [14], [15], a canonical right-handed helical filament with six RadA monomers per helical turn [16], [17], [18], [19], an overextended right-handed helical filament with three monomers per helical turn [20], [21] as well as a left-handed helical filament with four monomers per helical turn [22]. These crystal structures have added considerable understanding to homologous recombination.

A comparative structural analysis of different RadA polymers revealed that the majority of secondary structures in these structures are conserved, except that their NTDs and CADs undergo rigid body movements [22], [23]. We identified a hinge region, referred to as the subunit rotation motif (SRM), is responsible for transition between different RadA polymers. The SRM is located between the PM [i.e., Phe73 of *Sulfolobus solfataricus* (*Sso*) RadA] and the CAD, and uses the PM as a fulcrum to produce rotation along the central axis of the protein polymer. Accordingly, a progressive clockwise axial rotation can account for the structural transition from a protein ring (PDB accession code: 1PZN) to a right-handed RadA-AMP-PNP filament with six monomers per helical turn (PDB accession code: 1T4G), then to an overextended right-handed filament with three monomers per helical turn (PDB accession code: 2Z43), and, finally, to a left-handed filament with four monomers per helical turn (PDB accession code: 2DFL).

SRM-mediated axial rotation of RadA helical filaments might couple ATP binding and hydrolysis to homology pairing and strand exchange reactions [22], [23]. Arg83, an evolutionarily-conserved amino acid in the SRM of *Sso*RadA, controls the width of ATP binding pocket via salt-bridging with two negatively charged residues, Glu96 and Glu157. By comparing the width of ATP binding interface between two neighboring promoters, we proposed earlier that the 1T4G right-handed, 2Z43 overextended right-handed, and 2DFL left-handed filaments might represent the TP (ATP-bound), DP (ADP-P_i_ bound), and E (empty) states of RadA, respectively [22], [23]. Finally, a key consequence of these structural transitions is the progressive relocation of NTD dsDNA binding region and L1 ssDNA binding motif. For example, in the 2Z43 overextended right-handed filament, L1 relocates to the exterior surface of the filament and, and together with the NTD, constitutes an outwardly open palm structure that is wide enough to simultaneously accommodate a ssDNA and a donor dsDNA during the homologous pairing or search reaction [21]. Therefore, the 2Z43 overextended right-handed filament was considered to be a conformation during the homology pairing and search reaction. As described above, the RecA-ssDNA-ADP-AlF_4_^−^-Mg^2+^ nucleoprotein filament recently reported [11] is also overextended and responsible for homology pairing and search.

Taken together, these structural studies indicate that the NTDs and CADs are flexible to allow them to carry out rigid body movement around the central axis of helical filaments. To further substantiate the functional significance of axial rotation of RadA helical filament, it is necessary to determine new structures of RadA filaments as well as of RadA-DNA nucleoprotein filaments. In the present study, we report three new crystal structures of *Sso*RadA left-handed helical filaments [Protein Database (PDB) accession codes: 2ZUB, 2ZUC and 2ZUD]. These structures indicate additional structural flexibility of RadA helical filaments made possible by a hinge region between NTD and PM. We show, too, by mutant analysis that this hinge region is important for *Sso*RadA's function of promoting homologous recombination, particularly for the 2Z43 overextended right-handed filament.

## Results

### Overall structures

**Figure 1:**
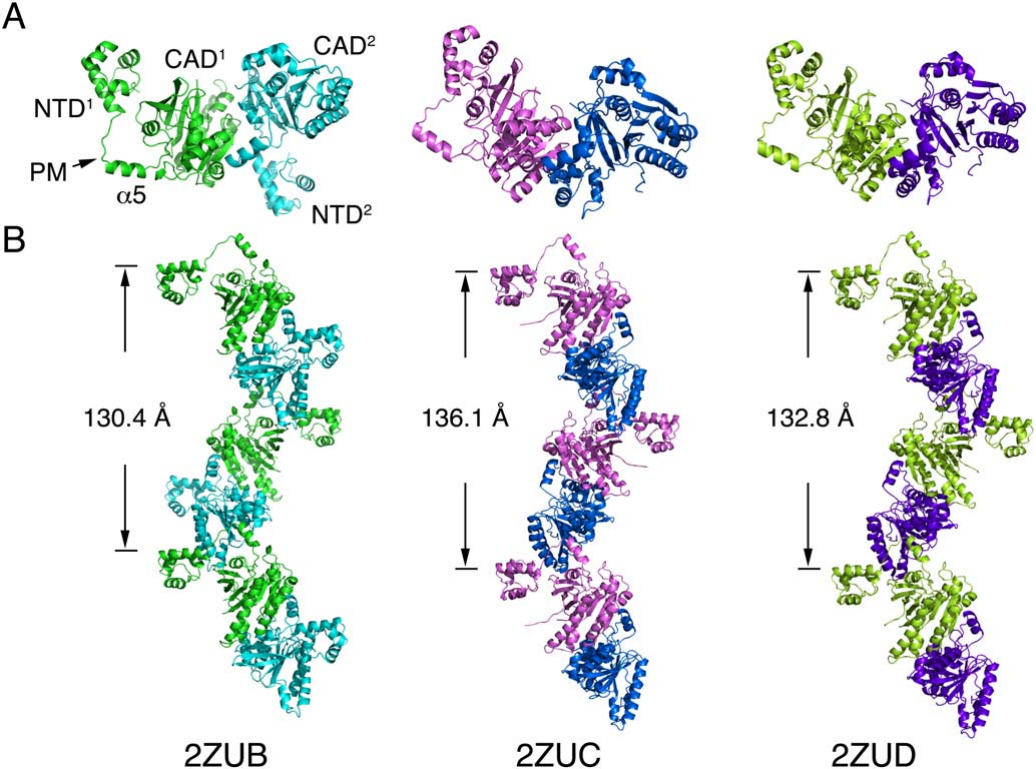
Crystal structures of three new left-handed RadA helical filaments. The ribbon representations of RadA dimers (A) and helical filaments (B). Each RadA promotor is indicated by different colors. The helical pitches and protein database accession codes of these three helical filaments are also indicated, respectively.

The first left-handed helical filament (2DFL) that we reported [22] has a helical pitch of 125.6 Å and contains 4 identical monomers per helical turn. Here we report additional three new crystal structures of left-handed helical filaments. Their helical pitches are 130.4 Å (2ZUB), 136.1 Å (2ZUC) and 132.8 Å (2ZUD), respectively (Figure 1). These crystals all belong to the space group *P2_1_2_1_2_1_* (Table 1). Unlike 2DFL, these three new left-handed helical filament structures are composed of two identical RadA dimers in each helical turn, and the two protomers in each dimer are structurally different. It was reported before that RecA family proteins might function as a dimer. First, the helical filament structure (PDB accession code 1SZP) of the yeast Rad51-I345T gain-of-function mutant suggested that the functional unit of Rad51 might be a dimer [16]. Second, a study of the *Ec*RecA fused dimer also indicated that dimeric RecA might be the functional unit for assembly of active nucleoprotein filaments and for their coordinated activities during homologous recombination [24]. Therefore, a dimeric functional unit could exist not only in right-handed filaments but also in left-handed helical filaments.

**Table 1:** Data collection and refinement statistics for the orthorhombic *Sso*RadA crystals, which belong to the space group *P2_1_2_1_2_1_*.

	2ZUB	2ZUC	2ZUD
**Data collection**			
Unit cell *a*, *b*, *c* (Å)	50.8, 103.5, 130.4	47.1, 114.8, 136.1	52.0, 115.1, 132.8
Resolution (Å)	30 – 2.9 (3.00 – 2.90)	30 – 3.3 (3.42 – 3.30)	30 – 3.2 (3.31 – 3.20)
Unique reflections	15861 (1560)	10381 (1027)	13493 (1316)
Redundancy	5.8 (5.9)	4.4 (4.1)	5.9 (6.0)
Completeness (%)	99.7 (99.6)	89.1 (90.2)	99.7 (99.8)
Average I / (I)	16.8 (2.7)	13.4 (1.9)	25.3 (5.0)
R_merge_ (%)	8.9 (50.4)	8.9 (56.6)	8.6 (47.4)
**Refinement**			
Number of reflections	15107 (1408)	9650 (779)	12930 (1065)
R_work_ (95% data)	0.224 (0.294)	0.227 (0.335)	0.225 (0.283)
R_free_ (5% data)	0.274 (0.310)	0.281 (0.413)	0.277 (0.394)
R.m.s.d bond distance (Å)	0.008	0.015	0.013
R.m.s.d bond angle (°)	1.3	1.6	1.4
**Ramachandran plot** (% residues)			
In most favored regions	85.0	87.8	87.2
In additional allowed regions	13.8	11.2	12.4
In generously allowed regions	1.0	0.6	0.0
In disallowed regions	0.2	0.4	0.4
Average B (Å^2^) / No. of non-H atoms			
Protein	49.1 / 4504	84.2 / 4661	55.0/ 4610
Water	42.3 / 115	59.5 / 12	42.4 / 77

Numbers in parentheses are for the highest resolution shells. All positive reflections were used in the refinement.

**Figure 2:**
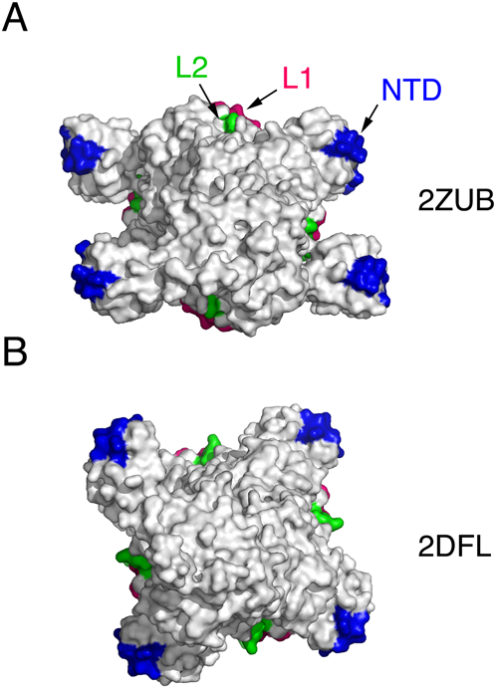
Top views of two left-handed RadA helical filaments. The ssDNA binding L1 and L2 motifs are highlighted in hotpink and green, respectively. The dsDNA binding region or N-terminal domain (NTD) are highlighted in blue.

A unique property of these left-handed helical filaments, as compared to other known structures of RecA family proteins [22], [23], is that their DNA binding motifs (i.e., L1, L2 and NTD) are all located at the outermost surface of helical filaments. Moreover, the NTDs are separated a long way from L1 and L2 motifs (Figure 2). We found that the locations of the L1 and L2 motifs in the four left-handed helical filaments are relatively conserved, but in contrast, the NTD locations are quite different. For example, the two neighboring NTDs in the 2ZUB helical filament are located at 180° to each other. Subsequently, in each helical turn, two NTDs face in one direction, and the other two face in the opposite direction (Figure 2A). By contrast, in the 2DFL, 2ZUC and 2ZUD helical filaments, each NTD was arrayed perpendicularly from the two neighboring NTDs. As a result, the four NTDs in a helical turn each face in a different direction (Figure 2B).

### Monomeric structures

**Figure 3:**
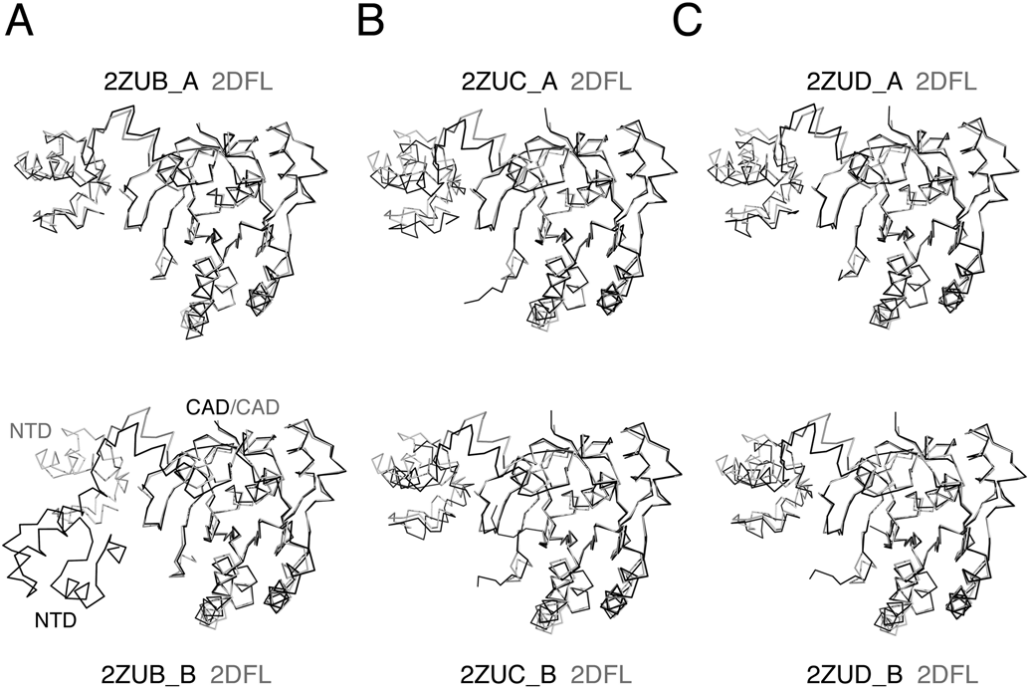
Monomeric structures of the three new left-handed helical filaments. Superposition of the ribbon representations of the monomer structure of 2DFL filaments (in grey) to those of 2ZUB, 2ZUC and 2ZUD filaments (in black), respectively. One protomer in each filament is almost identical in structure to that of 2DFL. They were assigned as 2ZUB_A, 2ZUC_A and 2ZUD_A, respectively. The other protomers were then assigned as 2ZUB_B, 2ZUC_B and 2ZUD_B, respectively.

We compared the two non-identical protomers in the three new filament structures to that of 2DFL by overlapping their main polypeptide chains, and found that one protomer in each is almost identical in structure to that of 2DFL. They were assigned as 2ZUB_A, 2ZUC_A and 2ZUD_A. The other protomers were then assigned as 2ZUB_B, 2ZUC_B and 2ZUD_B, respectively (Figure 3). Intriguingly, the NTD of 2ZUB_B, as compared to that of 2DFL, exhibited a large rigid body movement between NTD and CAD (Figure 3A, low panel). This rigid body movement can explain why the NTDs of 2ZUB filaments are located differently to those of 2DFL, 2ZUC and 2ZUD filaments. Notably, the crystal of 2ZUB, but not those of 2DFL, 2ZUC or 2ZUD, was grown in a buffer containing a hairpin DNA. NTD had been implicated before as a binding site of donor dsDNA [9], [21]. Although no electron density of this DNA was observed in the 2ZUB protein crystal, it is still of interest to speculate that this hairpin DNA may be one of the causes for such a large rigid body movement.

### Protomer-protomer interactions at the ATP binding sites

The ATP-binding interface between the two neighboring promoters of the 2DFL left-handed helical filament are more open than those of the 1T4G right-handed filament and the 2Z43 overwound right-handed filament [21]. The *CCP4* program [25] was then employed to determine the contact areas of two different ATP binding interfaces in the 2ZUB, 2ZUC or 2ZUC left-handed filaments. We found the contact areas of ATP binding interface of 2ZUC (2564 Å^2^ and 2570 Å^2^) and 2ZUD (2583 Å^2^ and 2560 Å^2^) are nearly identical to that of 2DFL (2543 Å^2^). On the other hand, those of 2ZUB are 2090 Å^2^ and 2375 Å^2^ respectively. Therefore, the ATP binding interfaces between two neighboring promoters in the 2ZUB filament are slightly more open than in the 2DFL filament. According to our earlier hypothesis [22], [23], if the 2ZUB structure does exist in a homologous recombination reaction, it may represent a conformation occurring after that of 2DFL.

### Two hinge regions between NTD and CAD are responsible for their rigid body movements

**Figure 4:**
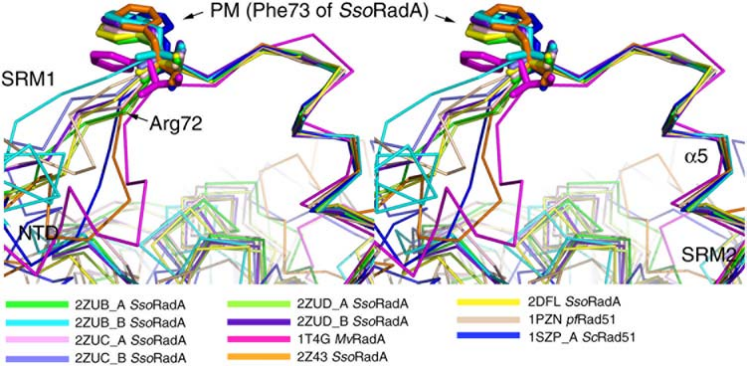
SRM1 is responsible for rigid body movements of NTD and PM in 11 different RadA/Rad51 quaternary structures. Stereo views of superposed structures at the SRM1-PM-SRM2 regions. The ribbon representation of each monomeric structure is shown in different color as indicated. The side chain of the hydrophobic resides in the PM (e.g., Phe73 in *Sso*RadA) are shown with the ball-and-stick models. NTD, SRM1, SRM2, α5 helices and Arg73 (in *Sso*RadA) are indicated by arrows, respectively.

We reported before that axial rotation of the SRM, a hinge region between the PM and the CAD, mediates progressive structural transitions from a protein ring, to the 1T4G right-handed filament, then to the 2Z43 overextended right-handed filament and finally to the 2DFL left-handed filament. Here, we found that another hinge region located between the NTD and the PM is responsible for a large rigid body movement of the NTD from the 2DFL structure to the 2ZUB structure (Figure 4). In both scenarios, the PM was used as a fulcrum to produce axial rotation of the NTD and the CAD along the axis of the helical filament. To further illustrate the structural flexibility between the NTD and the CAD, we compared 11 different RadA and Rad51 monomeric structures (i.e., 1PZN, 1T4G, 2Z43, 2DFL, 2ZUB_A, 2ZUC_A, 2ZUD_A, 2ZUB_B, 2ZUC_B, 2ZUD_B and 1SZP) by fixing their PMs and the neighboring α5 helix (Figure 4) and also by comparing their Φ and Ψ angles as described previously [22] (Table 2). The results indicate that structural changes of the two hinge regions are indeed responsible for different RadA quaternary structures. The first hinge region, located between the NTD and the PM, is referred to here as “subunit rotational motif 1 (SRM1)”. Accordingly, the hinge region located between the PM and the CAD (or immediately after the α5 helix) is referred to as “subunit rotation motif 2 (SRM2)” (Figure 4). The SRM2 was previously referred to as the SRM [22].

**Table 2:** The torsion angles (Φ and Ψ) of the SRM1 and PM amino acid residues.

*Mv*RadA 1T4G	59	60	61	62	63	64	65	66	67	68	69	70	71	72	73
a. a. sequence	L	C	D	L	**G**	F	K	S	G	I	D	L	L	K	Q
Φ	−64	−90	57	−93	−131	−80	−131	−156	60	−54	−63	−70	−70	−60	−63
Ψ	−34	0	51	73	−154	140	153	176	−143	−28	−24	−35	−39	−40	−46
*Sso*RadA 2Z43	68	69	70	71	72	73	74	75	76	77	78	79	80	81	82
a. a. sequence	A	L	D	I	**R**	F	K	T	A	L	E	V	K	K	E
Φ	−69	−101	63	−95	−170	−68	−121	−100	−57	−71	−63	−66	−59	−61	−67
Ψ	−25	9	37	110	169	125	174	155	−27	−42	−34	−46	−47	−39	−22
*Sso*RadA 2DFL	68	69	70	71	72	73	74	75	76	77	78	79	80	81	82
a. a. sequence	A	L	D	I	**R**	F	K	T	A	L	E	V	K	K	E
Φ	−48	−57	68	−117	−122	−87	−89	−118	−55	−86	−69	−48	−65	−75	−54
Ψ	−59	−17	85	88	132	111	−174	143	−10	−47	−55	−42	−23	−53	−47
*Sso*RadA 2ZUB_A	68	69	70	71	72	73	74	75	76	77	78	79	80	81	82
a. a. sequence	A	L	D	I	**R**	F	K	T	A	L	E	V	K	K	E
Φ	−61	−73	−12	−87	−124	−97	−101	−90	−54	−73	−58	−58	−66	−72	−49
Ψ	−70	19	113	135	132	107	168	152	−24	−45	−55	−41	−24	−67	−39
*Sso*RadA 2ZUB_B	68	69	70	71	72	73	74	75	76	77	78	79	80	81	82
a. a. sequence	A	L	D	I	**R**	F	K	T	A	L	E	V	K	K	E
Φ	−84	−86	−83	−93	−127	−101	−70	−116	−53	−68	−47	−59	−56	−54	−59
Ψ	−35	−49	169	123	147	109	−177	151	−23	−67	−36	−51	−44	−57	−59
*Sso*RadA 2ZUC_A	68	69	70	71	72	73	74	75	76	77	78	79	80	81	82
a. a. sequence	A	L	D	I	**R**	F	K	T	A	L	E	V	K	K	E
Φ	−56	−66	61	−100	−112	−101	−88	−79	−68	−69	−64	−74	−54	−68	−65
Ψ	−24	−31	76	110	134	97	161	165	−33	−44	−35	−44	−35	−44	−22
*Sso*RadA 2ZUC_B	68	69	70	71	72	73	74	75	76	77	78	79	80	81	82
a. a. sequence	A	L	D	I	**R**	F	K	T	A	L	E	V	K	K	E
Φ	−47	−67	−83	−86	−121	−90	−81	−70	−64	−51	−62	−71	−66	−66	−64
Ψ	−25	127	61	102	121	98	148	163	−53	−48	−37	−33	−34	−40	−33
*Sso*RadA 2ZUD_A	68	69	70	71	72	73	74	75	76	77	78	79	80	81	82
a. a. sequence	A	L	D	I	**R**	F	K	T	A	L	E	V	K	K	E
Φ	−68	−79	64	−82	−122	−82	−80	−84	−57	−70	−51	−70	−53	−61	−67
Ψ	−13	−27	60	121	117	82	166	153	−33	−57	−35	−50	−37	−46	−21
*Sso*RadA 2ZUD_B	68	69	70	71	72	73	74	75	76	77	78	79	80	81	82
a. a. sequence	A	L	D	I	**R**	F	K	T	A	L	E	V	K	K	E
Φ	−72	−69	69	−88	−124	−84	−81	−78	−56	−70	−60	−66	−56	−63	−69
Ψ	−17	−25	58	114	121	87	157	146	−32	−51	−34	−48	−36	−40	−20
*Sc*Rad51 1SZP	139	140	141	142	143	144	145	146	147	148	149	150	151	152	153
a. a. sequence	L	V	P	M	**G**	F	V	T	A	A	D	F	H	M	R
Φ	−87	−120	−57	−136	−146	−80	−163	−64	−65	−58	−55	−63	−34	−74	−45
Ψ	−26	115	125	58	177	167	148	161	−41	−47	−32	−67	−24	−69	−24

Torsion angles in the SRM and PM of different RadA/Rad51 filaments exhibiting significant changes (>60°) compared to those of the *Mv*RadA-AMPPNP right-handed filament are highlighted in red. R72 of *Sso*RadA and the corresponding residues in *Mv*RadA(G63) and *Sc*Rad51(G143) are highlighted in bold.

### Arg72 is functionally important for SsoRadA's recombinase activity

**Figure 5:**
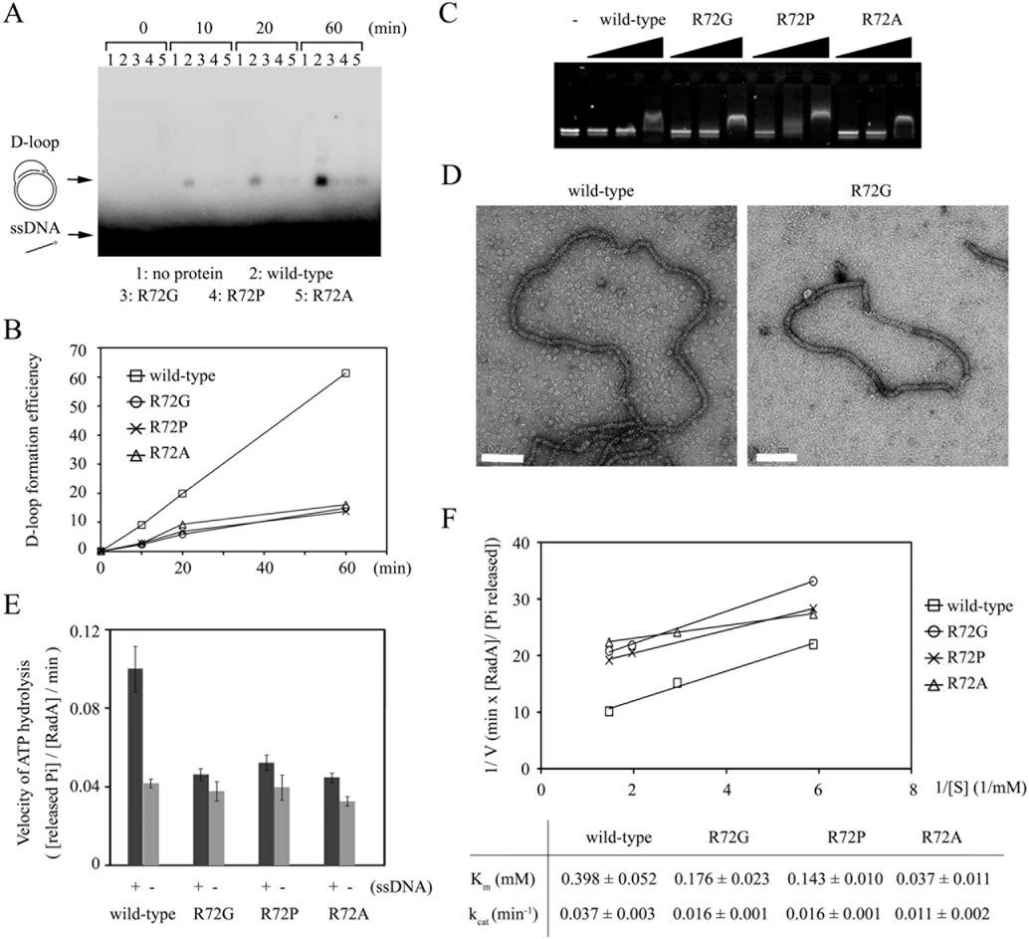
Functional characterizations of *Sso*RadA SRM1 point mutant proteins. All experiments were performed as described before [22], [26]. (A) Time course analysis of D-loop formation was carried out as described before. (B) Quantitation of the D-loop time course experiment shown in (A). (C) EMSA analysis. ΦX174 ssDNA (12 µM nucleotides) was incubated with 1, 2, or 10 µM *Sso*RadA protein at 65°C for 25 min. The reactions were analyzed by electrophoresis in 1.4% agarose gels with TAE buffer, stained with SYBR-Green II (Molecular Probes), and then visualization by UV illumination. (D) EM imaging results. Shown are nucleoprotein filaments of wild-type or R72G proteins with a ΦX174-ssDNA, respectively. Scale bars (in white) are 100 nm. (E) The ATPase activities of *Sso*RadA proteins in the presence (+) or absence (−) of ssDNA substrates. ATP hydrolysis was determined by release of inorganic phosphates (^32^Pi) from [γ-^32^P] ATP as described previously [22]. (E) Kinetic analysis of the ssDNA-stimulated ATPase activities of the wild-type and mutant *Sso*RadA proteins with different amount of [γ-^32^P] ATP. K_m_ and k_cat_ were also calculated as described before [22].

Arg83 of SRM2 is essential for *Sso*RadA's function of promoting both DNA-dependent ATPase activity and D-loop product formation [22]. Here, we also examined if SRM1 contains any critical amino acid residue that is functionally important for homologous recombination. We found that Arg72, a residue next to the PM (i.e., Phe73), is responsible for progressive rotation or movement of the NTDs in different RadA/Rad51 quaternary structures (Figure 4). To substantiate its functional significance, we expressed and purified three *Sso*RadA mutant proteins (R72G, R72P and R72A) in which Arg72 was replaced by Gly, Pro or Ala, respectively. These mutant proteins were then compared to the wild-type protein with various enzymatic assays. First, time course analysis of D-loop formation was carried out to determine if these SRM1 point mutants could catalyze the homologous strand assimilation reaction. As reported before, the wild-type *Sso*RadA catalyzed homology-dependent D-loop formation between a ^32^P-labeled oligonucleotide P1656 (50-mer) and a supercoiled plasmid, GW1 [22], [26]. We found that all three mutants (R72G, R72P and R72A) were defective in promoting D-loop formation (Figures 5A and 5B). Second, electrophoresis mobility shifting assay (EMSA) and EM imaging analysis were also performed to determine if these proteins could bind a virion ΦX174-ssDNA in the presence of AMP-PNP. The EMSA revealed that ΦX174-ssDNA migrated more slowly in an agarose gel as the concentration of wild-type or mutant RadA protein increased, indicating that these mutants possess equivalent or even greater capability to bind ssDNA (Figure 5C). EM imaging analysis also revealed that both wild-type and mutant proteins could form a nucleoprotein filament with a circular ΦX174-ssDNA (Figure 5D). Therefore, the defects of these three mutants are unlikely to be a result of protein polymerization or ssDNA binding.

RecA family proteins all hydrolyze ATP in response to ssDNA. We thus compared the ssDNA-stimulated ATPase activities of these three mutants, by monitoring the release of ^32^Pi from [γ-^32^P]ATP. The *K*_m_ of the wild-type *Sso*RadA for ATP in the ssDNA-stimulated ATPase activity assay was determined to be 0.398±0.052 mM, and *k*_cat_ (determined by the ratio of V_max_ to E, where E was the concentration of *Sso*RadA protein used) was 0.037±0.003 min^−1^. We found that the three mutants exhibited much lower ATPase activity in response to ssDNA. The *K*_m_s of R72G, R72P and R72A were 0.176±0.023 mM, 0.143±0.010 mM and 0.037±0.011 mM, respectively, while their *k*_cat_s were 0.016±0.001 min^−1^ (R72G), 0.016±0.001 min^−1^ (R72P), and 0.011±0.002 min^−1^ (R72A). Therefore, these three mutants exhibit a higher affinity to ATP, but are less effective in ATP hydrolysis (Figure 5E).

Taking together all of these biochemistry and EM results, we conclude that Arg72 has an important function in homologous recombination. Such a function likely occurs after a RadA-ssDNA-ATP nucleoprotein filament is assembled and is required for the nucleoprotein filament to catalyse ATP hydrolysis and D-loop formation.

## Discussion

For almost two decades, RecA family proteins were thought to exist as protein rings or as right-handed helical filaments. Recently, we reported the first crystal structure of *Sso*RadA left-handed filament (2DFL) [22]. An additional three new crystal structures of *Sso*RadA left-handed helical filaments (2ZUB, 2ZUC and 2ZUD) are presented here to substantiate the existence of left-handed helical filaments.

The three new structures have revealed two important structural properties. First, the basic structural element of these three new helical filaments is a RadA dimer. Similarly, a yeast Rad51-I345T gain-of-function mutant protein helical filament [16] also indicated that the function unit of RecA family protein is likely a dimer. Such a functional unit can exist not only in right-hand filaments but also in left-handed filaments. Second, the NTD of the 2ZUB filament undergoes a much larger rigid body movement than that of the 2DFL filament (Figure 2), indicating that the NTD is highly flexible. Subsequent structural and biochemical analysis revealed that SRM1, a hinge region between the NTD and the PM, is not only responsible for structural flexibility of the NTD but also is important for RadA's function in promoting D-loop formation.

**Figure 6:**
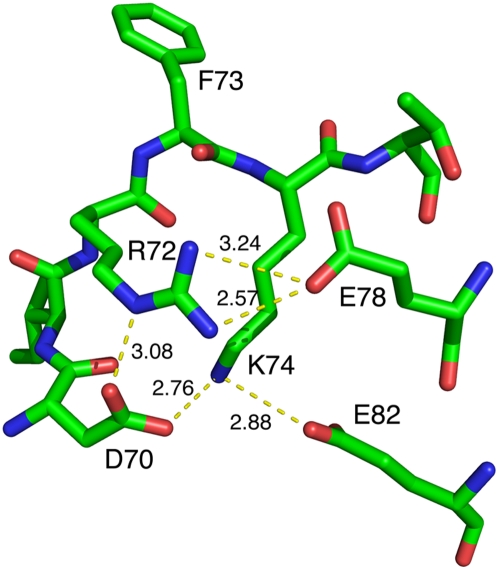
Arg72 is the central organizer for a salt-bridge network in the 2Z43 structure. Shown is the ball-and-stick model of a short peptide at the PM. The oxygen and nitrogen atoms are indicated in red and blue, respectively. Phe73 (F73) is the hydrophobic residue responsible for RadA protein polymerization. The salt-bridges are also shown by yellow dot line, and their distances (Å) are indicated in yellow. The side chain of Arg72 (R72) forms salt-bridges with those of Asp70 (D70) and Glu78 (E78), and the side chain of Lys74 (K74) also forms salt-bridges with those of Asp70 (D70) and Glu82 (E82), respectively.

We showed that Arg72, a key residue at SRM1, is required for an assembled RadA-ssDNA-ATP nucleoprotein filament to promote ATP hydrolysis and D-loop formation. According to the hypothesis we proposed previously [21], [22], [23], we believe Arg72 may function during a structural transition from the 1T4G RadA-AMPPNP right-handed filament to the 2Z43 RadA overextended right-handed filament. 2Z43 was considered to be a conformation of RadA-ADP-Pi filament [22], [23]. Accordingly, we speculate that Arg72 may have a unique structural role in the 2Z43 filament. Indeed, in the 2Z43 filament, Arg72 forms salt bridges with Asp70 and Glu78, respectively. Moreover, Asp70 and Glu82 also form salt bridges with Lys74 (Figure 6). These salt bridges may reinforce the movement of the NTD and L1 to the exterior surface of the helical filament, so that L1 and the NTD could constitute an outwardly-open palm structure to mediate homologous pairing between ssDNA and donor dsDNA [21], [22], [23]. Here, Arg72 serves as a central organizer of the salt bridge network in the 2Z43 filament. Intriguingly, this salt bridge network is not formed in any other RadA polymer structures described in this report (data not shown).

**Figure 7:**
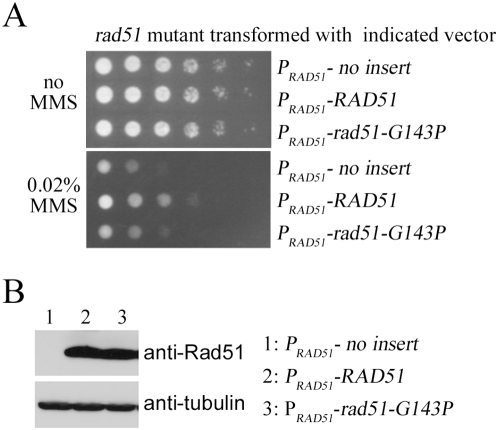
Structural flexibility of SRM1 is essential for yeast Rad51 function *in vivo*. The yeast *rad51* null mutant was transformed either with the empty control vector pYC2, or with the pYC2-Rad51 expression vectors for wild-type Rad51 or rad51-G143P proteins as indicated. Induction of these proteins was under the control of the *RAD51* gene promoter. (A) MMS sensitivity assay was carried out as described before (1). (B) Western blot analysis of the wild-type and G143P mutant proteins. Total cell lysates were separated by SDS-PAGE and then analyzed by Western blotting. Anti-Rad51 antibody (Santa Cruz Biotechnology) and anti-tubulin (Invitrogen) were used for detection of Rad51 and tubulin proteins. Tubulin was used here as a protein loading control. Final detection was performed using the ECL detection system, with the emitted chemiluminescence recorded on X-ray film.

Finally, an amino acid sequence alignment between various RecA family proteins revealed that the equivalent amino acids to Arg72 in other aracheal RadA proteins or in eukaryotic Rad51 and Dmc1 were glycines, including Gly143 of yeast Rad51. Gly143 is essential for yeast Rad51's function *in vivo*, because a yeast mutant strain expressing the mutant Rad51-G143P protein was sensitive to the DNA-damaging reagent methyl methanesulfonate (MMS). By contrast, the yeast strain expressing wild-type Rad51 proteins was MMS-resistant (Figure 7). Since a glycine residue allows more structural flexibility than proline, we assume that SRM1 of the Rad51-G143P mutant is more structurally rigid than that of the wild-type protein. However, additional biochemistry studies are needed to substantiate the biological relevance of SRM1, for example to verify if the Rad51-G143P mutant may exhibit other defects, such as in polymerization, ssDNA binding, or interaction with mediator proteins.

Together, the results of SRM1 mutants described in this study and those reported previously of SRM2 mutants [22], [23] suggest that during a homologous recombination reaction, structural flexibility of SRM1 and SRM2 is the key for progressive rigid body movement of the NTD and the CAD around the central axis of a helical filament. The energy of ATP hydrolysis is used to promote rotation of SRM1 and SRM2 along the central axis of RadA helical filaments. Such a point of view is consistent, at least partly, with those described before by other investigators [16], [27], [28].

Questions have been raised recently over the right-to-left axial rotation model we proposed earlier [22], [23]. First, earlier atomic force microscopy and EM imaging studies revealed that *Sso*RadA and yeast Dmc1 proteins could form both right- and left-handed filaments *in vitro* [22], [26], [29]. However, it is generally known that negative staining EM analyses cannot distinguish the handedness of a macromolecular filament [30]. Here, a virtual helical line was used to mathematically model the negatively-stained RecA family protein filaments [31]. We argued that such a virtual helical line could not physically represent a real RecA family protein filament negatively stained with uranyl acetate [32]. As compared to Egelman's virtual helical line, the latter not only is much thicker in diameter but also forms narrower helical grooves. As a result, the negative staining could not only mold around the helical filaments, outlining their structures, but also penetrate into helical grooves. Therefore, these helical grooves might be distinguished in the final two-dimensional EM image because they are accessible to the stains and harder for electrons to pass through. Due to the negative stains lying along these helical grooves, a projection EM image can reveal information about the helical handedness. By contrast, the helical grooves in Egelman's virtual helical line are too thin to enclose negative stains [see Figure 1 in 32]. Moreover, this virtual helical line also could not account for the observation that both right- and left-handed helices exist simultaneously in a nucleoprotein filament of RecA proteins and a relaxed circular dsDNA [see Figure 2 in 32]. Second, a recent EM imaging analysis reported that two eukaryotic RecA-like proteins (*i.e.*, yeast Dmc1 and Rad51 proteins) could only form right-handed nucleoprotein filaments with ssDNA or dsDNA, respectively [33]. In one deep-etch shadowing EM experiment, these authors used 1000 nucleotide ssDNA oligos or linear dsDNA to prepare the nucleoprotein filament but only presented very short (<10 helical pitches) and fragmented nucleoprotein filaments with right-handed helical pitches [See Figure 5 in 33]. Finally, the left-handed helical filaments were crystallized at a lower pH solution [22], and it was suggested, therefore, that this conformation might be an artifact of this particular crystallization condition. This concern may be addressed in the following way. As we proposed earlier, the left-handed filament represents a structural intermediate at the end of strand exchange reaction for ssDNA expulsion while holding onto a heteroduplex dsDNA. Without DNA substrate, the left-handed protein filament alone may be unstable at neutral pH condition. By contrast, it apparently becomes more stable at lower pH solution, because low pH mimics the effect of DNA binding as DNA is highly negatively-charged.

In conclusion, further study will be required to validate the existence and also the biological relevance of left-handed nucleoprotein filaments of RecA family proteins. Regardless, the ability of RadA or other RecA family proteins to form both right- and left-handed helices as well as toroids demonstrates the flexibility of this class of proteins. The results presented in this report indicate that such structural flexibility in RadA likely is mediated by rigid body movements between NTD and CAD, or in other words, by axial rotation of NTD and CAD along the central axis of RadA protein filament.

## Materials and Methods

### Protein purification, enzymatic assay, reagents and yeast mutant analysis

All biochemistry and genetic experiments were carried out as described previously [22], [26].

### Crystallization and data collection

*Sso*RadA proteins were crystallized using hanging drop vapor diffusion by mixing 2 µL of protein (16 mg/ml in 30 mM Tris-HCl, pH 8) with 2 µl of buffer C (30 mM Tris-HCl pH 8, 1 mM MgCl_2_, 1 mM AMP-PNP). For the 2ZUB crystal, a hairpin DNA (TG_4_T, CT_3_C_4_T_3_C, GA_10_G) was added to the protein solution at a molar ratio of 1∶1.4, and incubated at 65°C for 20 min. The crystals were obtained in about one month by adding 1∶1 (v/v) reservoir (2 M sodium formate, pH 4.6) to the protein-only or the protein-DNA solution.

X-ray diffraction experiments were conducted at Spring-8 in Japan and the National Synchrotron Radiation Research Center in Taiwan. Before flash cooling with liquid nitrogen, crystals were soaked in mother liquor containing 10% glycerol as a cryoprotectant. The diffraction data were processed and scaled using the HKL package [34]. The space group of the three crystals was found to be *P2_1_2_1_2_1_*, with slightly different unit cells (Table 1) In these three crystals, each asymmetry unit comprises two *Sso*RadA molecules. Although the three crystals were grown in the presence of AMP-PNP and/or a hairpin DNA, the nucleotide-binding site contained no corresponding electron density for AMP-PNP, Mg^2+^ ion, or hairpin DNA.

### Structural calculation and refinement

The structures of were determined by molecular replacement (MR) using the *CNS* program [35]. The search model was the C-terminal domain of *Sso*RadA left-handed protein filament (PDB ID = 2DFL). The N-terminal domains were assigned manually in the initial electron density maps, and the entire structures were reconstructed using the program *O* [36] and refined with *CNS*. Approximately 5% of the data were reserved for R_free_. The Ramachandran statistics for the final models were determined by *PROCHECK*. All structural figures were generated using *PyMol* (DeLano). The distance of translation and angles of rotation between different protomers in the protein filament were calculated by *O* and *CCP4* programs [25]. Data collection and refinement statistics are summarized in Table 1.

### Accession code

Atomic coordinates and structural factors have been deposited in the PDB with accession codes 2ZUB, 2ZUC and 2ZUD.
